# Mechanism of Anti-Cancer Activity of Curcumin on Androgen-Dependent and Androgen-Independent Prostate Cancer

**DOI:** 10.3390/nu12030679

**Published:** 2020-03-02

**Authors:** Nurul Azwa Abd. Wahab, Nordin H. Lajis, Faridah Abas, Iekhsan Othman, Rakesh Naidu

**Affiliations:** 1 Jeffrey Cheah School of Medicine and Health Science, Monash University Malaysia, Jalan Lagoon Selatan, Bandar Sunway, Selangor Darul Ehsan 47500, Malaysia; nurul.abd.wahab@monash.edu (N.A.A.W.); iekhsan.othman@monash.edu (I.O.); 2 Laboratory of Natural Products, Faculty of Science, Universiti Putra Malaysia, UPM, Serdang 43400, Malaysia; nordinlajis@gmail.com (N.H.L.); faridah@food.upm.edu.my (F.A.); 3 Department of Food Science, Faculty of Food Science and Technology, Universiti Putra Malaysia, UPM, Serdang 43400, Malaysia

**Keywords:** curcumin, androgen-dependent prostate cancer, androgen-independent prostate cancer, molecular mechanism, prostate cancer

## Abstract

Prostate cancer (PCa) is a heterogeneous disease and ranked as the second leading cause of cancer-related deaths in males worldwide. The global burden of PCa keeps rising regardless of the emerging cutting-edge technologies for treatment and drug designation. There are a number of treatment options which are effectively treating localised and androgen-dependent PCa (ADPC) through hormonal and surgery treatments. However, over time, these cancerous cells progress to androgen-independent PCa (AIPC) which continuously grow despite hormone depletion. At this particular stage, androgen depletion therapy (ADT) is no longer effective as these cancerous cells are rendered hormone-insensitive and capable of growing in the absence of androgen. AIPC is a lethal type of disease which leads to poor prognosis and is a major contributor to PCa death rates. A natural product-derived compound, curcumin has been identified as a pleiotropic compound which capable of influencing and modulating a diverse range of molecular targets and signalling pathways in order to exhibit its medicinal properties. Due to such multi-targeted behaviour, its benefits are paramount in combating a wide range of diseases including inflammation and cancer disease. Curcumin exhibits anti-cancer properties by suppressing cancer cells growth and survival, inflammation, invasion, cell proliferation as well as possesses the ability to induce apoptosis in malignant cells. In this review, we investigate the mechanism of curcumin by modulating multiple signalling pathways such as androgen receptor (AR) signalling, activating protein-1 (AP-1), phosphatidylinositol 3-kinases/the serine/threonine kinase (PI3K/Akt/mTOR), wingless (Wnt)/ß-catenin signalling, and molecular targets including nuclear factor kappa-B (NF-κB), B-cell lymphoma 2 (Bcl-2) and cyclin D1 which are implicated in the development and progression of both types of PCa, ADPC and AIPC. In addition, the role of microRNAs and clinical trials on the anti-cancer effects of curcumin in PCa patients were also reviewed.

## 1. Introduction

Prostate cancer (PCa) is the second leading cause of cancer-related deaths for males [1]. The incidence of PCa has significantly increased over the recent years [2]. In 2018 alone, there were 1.3 million new cases reported and 359,000 mortalities recorded worldwide. Other studies have reported PCa as the most commonly diagnosed cancer after lung and liver carcinoma in 105 countries, notably in the developed countries. A similar increasing trend has been noted in the United States of America (USA), across the years, from 164,690 cases in 2018, to 174,650 new cases in 2019. Furthermore, there were 29,430 deaths in 2018, while the estimated mortality in 2019 has increased to 31,620 [2]. Globally, by 2030, the PCa incidence is expected to rise to 1.7 million, together with 499,000 deaths [3]. In the USA, PCa has the highest prevalence, with more aggressive phenotype among African-American with 2.4 times higher mortality rate, compared to white men [4,5]. However, the justification for such evidence remains inconclusive. Besides, despite the low number of cases recorded in the past, the PCa incidence in Northeast Asia has increased in recent years, which is strongly associated with the economic development and westernised lifestyle [6]. 

PCa is known as a heterogenous disease. Malignant transformation of prostate cells occurs through multiple processes, initiating as prostatic intraepithelial neoplasia (PIN), followed by localized PCa and then progress to locally invasive adenocarcinoma, metastasise to distant sites, primarily to the lymph nodes or bone, and eventually develop androgen-independent phenotype [7]. PIN is a premalignant lesion and the most established precursor of prostatic carcinoma. PIN is associated with progressive abnormalities of phenotype and genotype changes, indicating impairment in cell differentiation and regulatory control with advancing stages of prostate carcinoma. Currently, PIN is only detected through a biopsy. The clinical importance of PIN is its high predictive value as a marker for adenocarcinoma and its identification warrants repeat biopsy for concurrent invasive carcinoma [8]. The risk factors for PCa are numerous and heterogeneous which could be age, sedentary lifestyle, family history, environment, ethnic factors and genetics [9]. Moreover, a high protein diet or dairy product-based calcium intake may elevate the risk of the disease [10]. Accordingly, changes in diet and lifestyle, specifically exercise and smoking cessation, may reduce PCa progression. Phytochemicals, vitamins and some minerals that are found in edible plants such as vitamin A, isoflavones (e.g., genistein and daidzein), vitamin E, lycopene, selenium may also minimize the risk of subsequent cancer recurrence, progression, or death due to PCa [11,12,13]. 

A few current treatment options are shown successful in treating localised and androgen-dependent PCa (ADPC) including hormonal treatment, surgery and radiotherapy. However, over time, these cancerous cells progress to androgen-independent PCa (AIPC) which continuously grow despite hormone depletion. At this particular stage, androgen depletion therapy (ADT) will be no longer effective as these cancerous cells are hormone-insensitive and capable of growing in the absence of androgen, leading to metastasis [14,15]. Furthermore, the current therapy for AIPC is mainly palliative, in the form of taxane-based therapy, cytotoxic agent (e.g., docetaxel/predisone), novel hormonal therapies which target AR signalling (e.g., abiraterone acetate and enzalutamide regimes), radiotherapy for reduction of bone metastases (e.g., radium-223), immunotherapy (e.g., sipuleucel-T) and treatment by combination of different drugs [16]. Furthermore, the effectiveness of these drugs remains short-term, yielded adverse effects, and there is no evidence that these drugs could increase life expectancy [17,18]. Thus, numerous laboratory investigations and clinical studies have focused in identifying other novel potent anti-cancer molecules and relevant pathways to address AIPC occurrence, to extend life expectancy and improve health-related quality of life in patients with PCa.

The cost of prostate cancer care and available drugs can be high, thus limiting its potential widespread usage in less developed countries. Hence, it is urgent to develop a safe, potent, affordable, easily manufactured form of novel drug to treat AIPC. Throughout recent years, there is an emerging trend of using natural products from fungi, plants, and animals, for medicine, primarily due to their beneficial bioactive compounds as well as their lower toxicity [19]. The use of medicinal plants for health well-being has increased tremendously across the world. Indeed, more than half of the current drugs that available nowadays are originally obtained from such natural products.

## 2. Mechanism of Progression from Androgen-Dependent to Androgen-Independent Prostate Cancer

**Figure 1:**
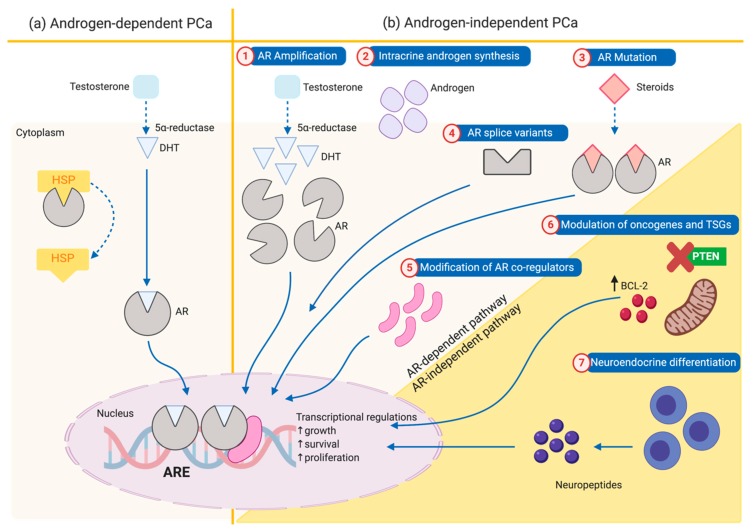
Molecular mechanisms of (**a**) androgen-dependent PCa (ADPC) and (**b**) development of androgen-independent prostate cancer (AIPC). The mechanism of the development of AIPC has been categorised based on AR-dependent that involving AR which include; (**1**) AR amplification, (**2**) intracrine androgen synthesis, (**3**) AR mutation, (**4**) AR splice variants and (**5**) modulation of AR co-regulators, (**6**) modulation of oncogenes and TSGs and (**7**) neuroendocrine differentiation. TSGs: Tumour suppressor genes; HSP: heat shock proteins; ARE: androgen response element; DHT: dihydrotestosterone; PTEN: phosphatase and tensin homologue deleted on chromosome-10.

**Figure 2:**
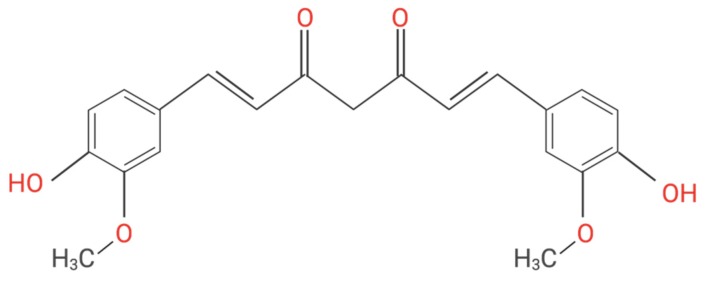
Molecular structure of curcumin. Curcumin is a symmetric molecule with chemical formula C_21_H_20_O_6_ and molecular weight 368.38. It consists of three chemical entities in its structure: two aromatic ring systems containing o-methoxy phenolic groups, linked by a seven-carbon linker consisting of an α, β-unsaturated β-diketone moiety.

Almost all PCa begins as ADPC, where the growth depends on androgen-induced androgen receptor (AR), as illustrated in Figure 1a [20,21]. Initially, ADT is effective in treating ADPC and still remains the foundational treatment of PCa. Unfortunately, nearly all PCa patients will eventually progress to AIPC which are currently incurable [22,23]. Several studies have shown that persistent AR signalling and aberrant AR expression are key contributing factors that support the progression to AIPC [24,25,26]. The augmentation of AR-mediated signals in AIPC cells resulted in increased cell proliferation despite of low testosterone levels and/or absence of androgens [27,28,29]. As shown in Figure 2b, the mechanisms contributed to the progression of AIPC are AR amplification, AR mutation, AR-splice variants, modification of AR co-regulators, prostate intracrine androgen biosynthesis, modulation of oncogenes and tumour suppressor genes, and neuroendocrine cells differentiation. The first five pathways are AR-dependent pathways which requires continuous activation of AR signalling. The latter two pathways are AR-independent pathways that do not involve AR signalling [17,30].

AR amplification and overexpression have been implicated in many AIPC cases, both in vitro and in vivo [31,32]. An aberrant gene amplification may lead to overexpression of AR and subsequently enhance AR-androgen ligand binding even at presence of low circulating androgen [25]. Additionally, PCa cells may also contain AR mutations. These mutations alter AR ligand-binding domain thus increase AR transactivation activity by increasing the binding specificity to other endogenous steroid ligands (e.g., progesterone, corticosteroids, and oestrogen) [33]. AR splice variants (ARVs) have also been found to be correlated with persistent AR activity and contribute to therapy resistance. Tumour cells harbouring ARVs lack of ligand-binding domain (LBD), enable cells to bypass the need of androgens because AR can become constitutively active [34]. It was also reported that intracrine biosynthesis of androgens from adrenal steroids and cholesterol also contribute to the elevated testosterone level in AIPC. Consequently, the presence of intracellular androgens is sufficient to activate the AR signalling [35]. The ratio between co-activator and co-repressor may also influence the overall AR activation in AIPC. Co-activators are overexpressed in AIPC cells and modulate AR activity allowing to gain androgen-independence [36]. In contrast, the co-repressor proteins have been reported to be down-regulated in AIPC, resulting in increased AR-mediated transcriptional activity [30].

The progression of PCa cells to neuroendocrine differentiation of PCa cells (NEPC) has led to treatment resistance and ultimately results in the progression to AIPC [37]. In contrast to the pathways discussed earlier, this pathway is categorised as AR-independent pathway. Despite of the absence of androgen, NEPC cells continually secrete neuropeptides such as serotonin and bombesin, which pose paracrine effects on the neighbouring cells, stimulating the proliferation, motility, and thus the metastatic potential of PCa cells [38]. Additionally, AIPC tumour cells develop the ability to survive in androgen castration via modulation of Bcl-2 oncogene and PTEN (phosphatase and tensin homologue deleted on chromosome-10) tumour suppressor genes. The pro-survival protein Bcl-2 has been reported to be highly expressed in PCa cells, and closely correlated with the progression of ADPC to AIPC growth state, although the mechanism is still uncertain [39]. Another important molecule, phosphatase and tensin homolog deleted on chromosome 10 (PTEN) expression has been observed to be downregulated in many cases of AIPC. Commonly, PTEN loss of function occurs in many types of cancers through various genetic alterations like deletion, mutation and methylation [40]. The loss function of PTEN leads to constitutive activation of PI3K pathway associated with cell proliferation, survival and migration. Contrarily, PTEN negatively regulates the activity of this pathway thus promoting apoptosis and inhibiting cell proliferation. It is hypothesized that PTEN loss or PI3K pathway activation stimulates the AR nuclear translocation and AR-mediated transcriptional activity [41]. 

## 3. Curcumin as a Potential Anticancer Agent for Prostate Cancer

Turmeric is derived from *Curcuma longa* rhizomes, has been used since ancient times for medical purposes for the treatment of various ailments and diseases [42]. Curcumin, known as diferuloylmethane, is the principal polyphenol of turmeric, responsible for its therapeutic effects [43,44]. Curcumin consists of two aromatic ring systems containing o-methoxy phenolic groups, connected by a seven carbon linker consisting of an α,β-unsaturated β-diketone moiety (Figure 2) [45]. There are numerous in vitro and in vivo, as well as clinical trials findings reporting the therapeutic efficacy of curcumin in treating many diseases since it exhibits anti-inflammatory, antioxidant, antibacterial, anti-fungal, and antiviral properties [46,47,48,49,50,51].

Curcumin is identified as a highly pleiotropic compound capable of influencing and modulating a diverse range of molecular targets, by altering cells’ gene expression and signalling pathways. Due to multiple-targeting characteristic, curcumin is able to regulate a diverse array of transcription factors, inflammatory cytokines, enzymes, kinases, growth factors, receptors, and apoptosis proteins that are frequently dysregulated in cancer. There are numerous pre-clinical and animal studies which conclude that curcumin as a potent anti-tumour agent, for its effectiveness in regulating several biological pathways which are implicated in tumorigenesis [52]. Curcumin interferes cancer growth by targeting a different multistep molecular tumorigenesis including tumour initiation and progression phase in a wide range of tumour cells [53,54]. Therefore, it possesses chemopreventive effects by reverse, suppress, prevent carcinogenesis and cancer progression. Several animal studies have shown that curcumin has a dose-dependent chemopreventive effect in different type of cancers, including PCa [55]. It was also reported that consuming curcumin could decrease the risk of PCa development [56]. Apart of its anti-cancer properties, curcumin also acts as a potent chemo- and radio-sensitiser agent [57,58,59]. Furthermore, curcumin has been proven safe for medical purposes, with low toxicity and fewer side effects regardless of the dosage consumed [60]. Clinical studies investigating curcumin’s safety and efficacy have supported that curcumin have a safe profile [46,61]. Moreover, curcumin has been categorised as Generally Recognised As Safe (GRAS) by the U.S. Food and Drug Administration (USFDA), with recommended serving dose ranging from 8 g/day to 12 g/day [62,63,64]. 

The first evidence of the anti-cancer properties of curcumin was published in 1985 [65]. Since then, a large amount of research exploring the effects of curcumin in cell lines, animal and human models have been conducted worldwide [66,67,68,69,70,71,72,73,74,75,76,77,78,79,80,81,82,83,84]. There are plenty of data on curcumin’s anti-tumour effects in many other types of cancer, however, evidences regarding the mode of actions of curcumin in PCa are considered limited [85,86,87,88]. For the evaluation of curcumin activity in in vitro model of PCa, the commonly used cells which represents ADPC is LNCaP cells, while PC-3 and DU 145 cells represent AIPC [89,90]. At the molecular level, curcumin inhibits the over-expression of oncogenes Bcl-2, AR signalling, epidermal growth factor receptor (EGFR), human epidermal growth factor receptor 2 (HER2), Cyclin D1, cyclooxygenase (COX-2), matrix metalloproteinase (MMP), protein kinases B (Akt), transcription factors (e.g., nuclear factor kB (NF-κB), activator protein 1 (AP-1), signal transducer and activator of transcription 3 (STAT3), and several signalling pathways like PI3K/Akt/mTOR, JAK/STAT and MAPK [91,92]. Previous studies have reported that curcumin demonstrates inhibitory growth effects on both ADPC (LNCaP) and AIPC (PC-3) cells, whereby its efficacy is comparable to conventional chemotherapeutic drugs [52,93,94]. Curcumin also significantly delays tumour growth and induces cell death in ADPC (LNCaP), AIPC (DU145) cells and (AIPC) PC-3 xenograft models [56,94,95,96]. Curcumin treatment also demonstrates strong selectivity towards prostate malignant cells over normal human prostate epithelial cells [97]. Hence, this review will discuss the curcumin’s mode of mechanisms as a potential anti-cancer agent influencing the key molecular targets and pathways which could offer an alternative in improving therapeutic strategies of PCa.

## 4. Selected Molecular Targets Effected by Curcumin in Prostate Cancer

**Figure 3:**
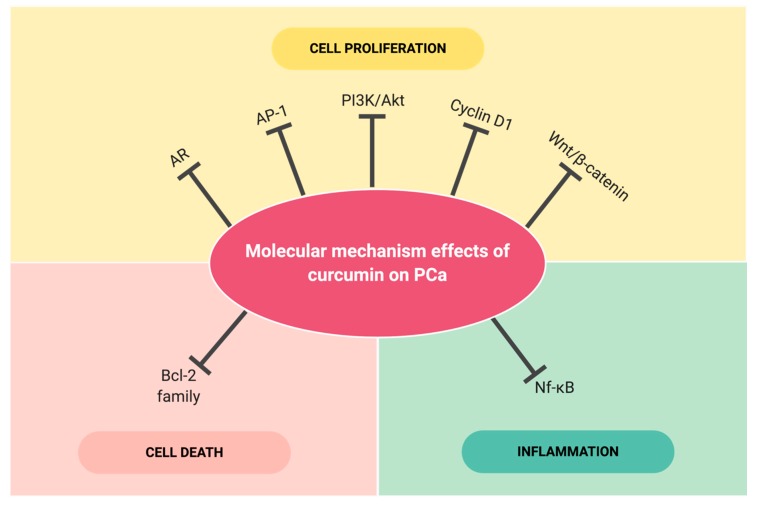
The key molecular targets of curcumin linked with inflammation, cell death, and cell proliferation in in vitro and in vivo models of PCa. The sign ⊣ indicated inhibition by curcumin. AR: Androgen receptor signalling; AP-1: Activating protein-1; PI3K/Akt/mTOR: Phosphatidylinositol 3-kinases/the serine/threonine kinase; Wnt/ß: Wingless (Wnt)/ß-catenin signalling, and molecular targets: NF-κB; Nuclear factor kappa-B; Bcl-2: B-cell lymphoma 2 and Cyclin D1.

The aberrant signalling pathways as well as the alteration of molecular targets in prostate tumorigenesis which lead to abnormal cell proliferation, cell survival, angiogenesis and metastasis are well-documented [98]. Studies have described the ability of curcumin to suppress the prostate carcinoma cells by interacting with different molecular targets such as p53, Ras, PI3K/Akt, Wnt-β catenin and mTOR [99]. The following sections will discuss the mode of actions of curcumin in targeting aberrant key molecules and signalling pathways in PCa. Based on in vitro, in vivo and clinical studies on PCa, several major signalling pathways and molecular targets of curcumin have been identified including AR, NF-κB, AP-1, PI3K/Akt, Bcl-2 family, Cyclin D1, and Wnt/ß-catenin as presented in Figure 3, will be discussed in detail as follows. Table 1 shows the summarize of molecular mechanism targeted by curcumin in vivo and in vitro against androgen-dependent PCa and androgen-independent PCa.

### 4.1. Androgen Receptor (AR)

Androgens, as well as AR signalling, are crucial in the development and function of male reproductive organs [137,138,139]. Androgens function predominantly by binding to AR, regulates a variety of cellular processes like cell growth and proliferation, signal transduction and protein folding [140]. The activation of AR signalling governs the PCa cells growth and is known to be a key driver for PCa tumorigenesis as well as an important factor for the progression to AIPC [140]. In the absence of androgens, AR remains inactive and sequestered in the cytoplasm by the chaperone super-complex from the heat shock protein (Hsp) family. Upon binding with 5α-dihydrotestosterone (DHT) which is an active metabolite of testosterone, AR dissociates from Hsp and undergoes conformational changes. The AR-DHT complex translocates to the nucleus, binds to the androgen response elements (AREs) resulting in the transcription and translation of the target genes, hence activates the AR signalling pathway [141].

The AR signalling pathway is one of the most common pathways dysregulated in PCa, which is reported higher prevalence in AIPC, denoted by a high level of prostate-specific antigen (PSA) [140,142]. There are several factors that lead to activation of AR despite the absence of constitutive androgens including synthetisation of steroids from adrenal glands, over-expression of AR co-activators, AR gene amplification or ligand-independent activation of AR by growth factors, cytokines, and steroids other than androgens [143,144,145]. This indicates that despite ADT, AR remains functional and AIPC development is still dependent on androgen-driven activity [146,147,148,149]. A persistent AR activation in AIPC leads to uncontrolled cell proliferation and metastasis, and subsequently resulting death in PCa patients [24]. 

**Figure 4:**
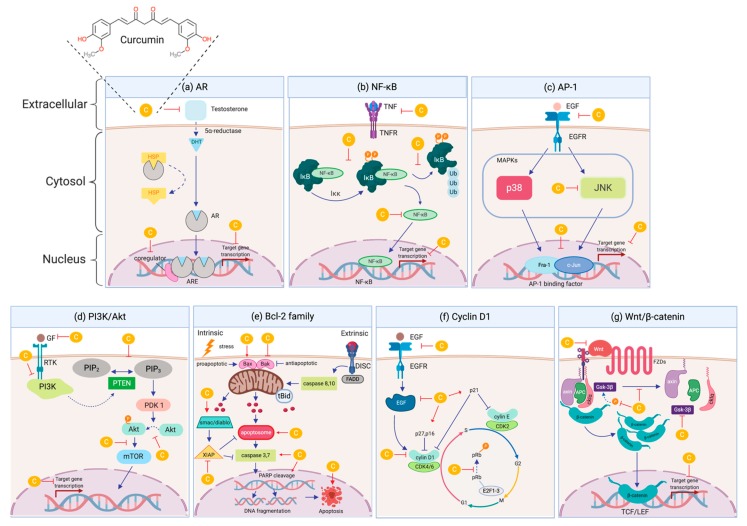
Mode of actions of curcumin as anti-cancer agent on the key molecular targets in aberrant signalling pathways of PCa. Curcumin exhibits anti-cancer properties by inhibiting signalling pathways and molecular targets; (**a**) Androgen receptor (AR) signalling; (**b**) Nuclear factor kappa-B (NF-κB); (**c**) Activating protein-1 (AP-1); (**d**) Phosphatidylinositol 3-kinases/the serine/threonine kinase (PI3K/Akt); (**e**) B-cell lymphoma 2 (Bcl-2); (**f**) Cyclin D1 and (**g**) Wingless (Wnt)/ß-catenin signalling. Molecular targets and signalling pathways that are induced by curcumin are noted by using →, while the inhibition represented by ⊣ symbol.

Curcumin as an anti-inflammatory agent, has the capability to suppress AR at the protein as well as AR gene transcription level in PCa cells [105,116]. The effects of curcumin on AR signalling are shown in Figure 4a. In response to curcumin, AR expression was downregulated in ADPC (LNCaP) cells by limiting the binding activity to the ARE of the PSA gene thus reducing its expression level [100,101]. Other studies have also reported that curcumin significantly inhibits cell proliferation and cancer cells growth in dose-dependent manner when treated in ADPC (LNCaP) cells via modulation of AR and its signalling pathway [102]. Curcumin also inhibits tumour growth and suppresses the PSA levels which stimulated by the activation of AR and interleukin-6 in ADPC (LNCaP) cells [100]. Nakamura *et al*., (2002) demonstrated that curcumin downregulates AR expression and transcriptional activity not only in ADPC (LNCaP) but also in AIPC (PC-3) cells [103]. Besides, curcumin was shown capable of decreasing intracellular prostate testosterone level in ADPC (LNCaP) cells and in transgenic adenocarcinoma of the mouse prostate (TRAMP) model, thereby prevent the activation of AR by downregulating the expression of steroidogenic acute regulatory proteins, CYP11A1 and HSD3B2 [102,150]. Moreover, curcumin treatment against AIPC (PC-3) cells also alter the over-expressed heat shock protein (Hsp90), resulting in the reduction of AR availability [104]. Curcumin also shows positive outcomes when tested in animal models, where it delays the tumour growth and suppresses AR expression in ADPC (LNCaP) xenograft model [105]. Another study on LNCaP xenografts models showed that curcumin inhibits AR through the modulation of Wnt/ß-catenin signalling [106]. 

An increased activity of AR coupled with the upregulation of a subset of AR-related co-activators mainly AP-1, NF-κB, and cAMP response element-binding protein (CBP) and co-activator protein p300 have contributed to the aggressiveness of the PCa disease and the progression to AIPC. Curcumin has the ability to downregulate the activation of AR-related cofactors when treated in ADPC (LNCaP) and AIPC (PC-3) cells [100,103]. Besides, other studies demonstrated that curcumin initiates apoptotic process accompanied with the downregulation of AR activity upon its treatment in ADPC (LNCaP) cells [93]. 

Curcumin also has shown the ability to mediate AR signalling by inhibiting NKX3.1, resulting in the downregulation of AR expression and DNA binding activity with ARE [151]. NKX3.1 is an androgen-adjusted NK-class homeobox gene, encodes a home-box-containing transcription factor that functions as a negative regulator of epithelial cell growth in prostate tissue [152]. The loss of NKX3.1 expression together with PTEN is constantly occurring in PCa, which therefore, is regarded as the key factor for normal organogenesis and carcinogenesis [107,153]. It was demonstrated that curcumin treatment in ADPC (LNCaP) cells has shown a reduction of NKX3.1 and AR expression [107]. Since activation of AR signalling has appeared to be the selective driving force for the development of ADPC and AIPC cells, the suppression of androgen/AR signalling is beneficial in PCa therapies. Curcumin has been shown is able to reduce PCa growth and malignancy by inhibiting the AR signalling and appear as a promising therapeutic target for PCa treatment.

### 4.2. Nuclear Factor kappa-B (NF-κB)

Nuclear factor kappa-B (NF-κB) is a pleiotropic transcription factor responsible for regulating cell signalling and various biological processes such as immune response, inflammation, cellular transformation, cell proliferation, angiogenesis, invasion and metastasis. The constitutive activation of NF-κB has been detected in many human malignancies [92]. In unstimulated cells, NF-κB present in inactive state binds to IκB proteins, preventing its translocation into the nucleus. Otherwise, the factor can be activated in response to a large variety of stimuli such as growth factor, protein kinases, oxidative stress inducers, mitogens, pro-inflammatory cytokines and chemokines (TNF-α, IL-1, IL-8, IL-6, CXCL12), and environmental stress factor [154,155]. Upon stimulation, IκBs are phosphorylated by the IκB kinase complex (Iκκ), including Iκκα, Iκκβ, and Iκκγ (NEMO); then, leads to ubiquitination and proteasome degradation. The phosphorylated NF-κB that dissociated from the Iκκ then bind to the target DNA gene promoter region, resulting in cell proliferation induction, metastasis, apoptosis suppression and treatment resistance [156,157]. 

Aberrant NF-κB activity has been associated to several types of carcinomas, including in human PCa cells and xenografts [158,159]. During prostate carcinogenesis, NF-κB promotes cancer cell survival, invasiveness, angiogenesis, metastasis, and chemo-resistance by inducing pro-survival genes (e.g., Bcl-2 and Bcl-xL), pro-inflammatory cytokines, growth factors such as vascular endothelial growth factor (VEGF), urokinase-type plasminogen activator (uPA), and matrix metalloproteinase-9 (MMP9) [160]. In addition, activated NF-κB upregulates tumour promoting cytokines, leads to increase AR activity in the androgen depletion state [159]. Several studies indicate that NF-κB plays an important role in regulating PCa transformation of ADPC to AIPC [161,162]. Other studies also reported an elevated expression of NF-κB in AIPC cells [163,164]. 

Basically, NF-κB proteins comprises of five different family members including NF-κB1 (p50/p105), NF-κB2 (p52/p100), RelA (p65), RelB, and c-Rel. These molecules can dimerise to form all possible combinations of homo and heterodimers. Among the NF-κB family subunits, NF-κB2 (p52/p100), RelA, RelB, and c-Rel have described are implicated in PCa [165]. Other studies reported that RelA (NF-κB/p65) is constitutively activated in human PCa and transgenic TRAMP [166]. Similarly, the upregulation of Iκκ activity has been observed alongside an increased IκBs protein phosphorylation and degradation, correlated with PCa [167]. Findings of earlier studies have indicated the wide range of anti-cancer and anti-inflammatory effects of curcumin attainable via suppressing the NF-κB activity. A number of studies indicated that curcumin as a potent inhibitor of NF-κB activation, works to suppress angiogenesis, invasion, and metastasis in various cancer cells, including PCa [168,169]. Following NF-κB inhibition, the cancer-related genes like Bcl-2, Bcl-xL, cyclin D1, IL-6, COX-2 and MMP9 are subsequently downregulated [170,171]. In many cases, curcumin prevents the NF-κB activation induced by abundance of agents through the inhibition of the upstream kinase active, namely Iκκα and Iκκβ, which are essential for the phosphorylation of IκBα protein [155]. These inhibitory actions are attributable to the sequential suppression of IκBα kinase activity, IκBα phosphorylation, IκBα proteasomal degradation, p65 phosphorylation, p65 nuclear translocation, and p65 acetylation [172,173,174]. Other researchers have underlined the link between curcumin-induced proteasomal malfunction with anti-inflammatory activities associated in the NF-κB pathway [56]. 

There are studies indicate the role of NF-κB in the survival of PCa cells, whereby curcumin is able to suppress NF-κB expression thus abrogates their survival mechanisms in both ADPC (inducible LNCaP) and AIPC cells (constitutive DU145), as shown in Figure 4b [92]. Curcumin also exhibits excellent anti-cancer activity by inhibiting cell proliferation and inducing apoptosis in AIPC (PC-3) cells, which probably contributed with the inhibition of NF-κB [108]. Inhibition of NF-κB signalling also restores responsiveness of AIPC cells to anti-androgen treatment [164]. Besides, NF-κB has also been implicated in the regulation of the cell cycle regulatory components involved in PCa. Curcumin on the other hand, downregulates cyclin D1 by inhibiting the activation of NF-κB, therefore subsequently suppress the cell proliferation in ADPC (LNCaP) cells [92]. Furthermore, curcumin also suppresses both the constitutional and TNF-α-induced NF-κB activation in AIPC (PC-3) cells, which contributes to the enhancement of cytotoxicity in the treatment combining curcumin and chemotherapeutic agents [109]. Curcumin is also reportedly downregulates CXCL-1 and -2 by targeting NF-κB signalling, simultaneously preventing metastasis in orthotopic mouse model of AIPC (PC-3) cells [110].

The combination of curcumin with tumour necrosis factor-related apoptosis-inducing ligand (TRAIL), a potent anti-cancer and inducer of apoptosis, have become an adjuvant therapy to improve the management of PCa disease, specifically AIPC. Impeded NF-κB action following curcumin treatment either as a stand-alone therapy or in combination with TRAIL against PCa cells resulted in suppression of angiogenesis, invasion, and metastasis. Previous studies have reported that ADPC (LNCaP) and AIPC (PC-3 and DU145) cells are either resistant or poorly susceptible to TRAIL therapy. However, a treatment of curcumin at certain concentrations is capable to sensitise these cancerous cells towards TRAIL-induced apoptosis [111,112]. Other studies reported that curcumin treatment in ADPC (LNCaP) cells initiates the induction of apoptosis by effecting both intrinsic and extrinsic pathways [111]. The mechanism by which curcumin augments TRAIL-induced cytotoxicity in ADPC (LNCaP) cells was shown to inhibit NF-κB by inhibiting phosphorylation and degradation of IκBα [114]. On the other hand, in AIPC cells, curcumin with the combination of TRAIL inhibits Akt-regulated NF-κB and NF-κB-dependent anti-apoptotic proteins such as Bcl-2, Bcl-xL, and X-chromosome-linked inhibitor of apoptosis protein (XIAP) [113]. Other studies reported that the mechanism by which curcumin sensitises both ADPC and AIPC cells to TRAIL therapy is attributed by inhibition of a constitutively active NF-κB, AP-1 and active anti-apoptotic Akt (p-Akt) [112,113,114]. In addition, the effect of curcumin combination with TRAIL was effectively inhibits the growth of AIPC (PC-3) tumour xenograft model, indicating the inhibition of NF-κB and AP-1 [115]. These findings suggest that combined curcumin/TRAIL chemo-immunotherapy may be a beneficial adjunct to the standard therapeutic regimens for PCa. To conclude, the aberrantly activated NF-κB signalling in PCa has been correlated with the progression of the disease including gaining aggressive phenotype, PSA recurrence, metastatic spread and chemoresistance. On the other hand, curcumin has been shown to be a potent inhibitor of transcription factors NF-κB, resulted in reduction of tumour growth, therefore has become a promising therapeutic target against PCa.

### 4.3. Activating Protein-1 (AP-1)

The activating protein-1 (AP-1) is a transcription factor, composed of dimer combinations primarily formed between basic leucine zipper family. The protein families belongs to Jun (e.g., c-Jun, JunB, and JunD); Fos (e.g., c-Fos, FosB, Fra-1, and Fra-2); activating transcription factor (ATF) (e.g., ATF2 and LRFI/ATF3); and musculoaponeurotic fibrosarcoma (MAF) (e.g., c-Maf, MafB, MafA, MafG/F/K); whereby all groups bind to a common DNA site, namely AP-1 binding site [175]. Conceptually, AP-1 proteins form homo- or/and heterodimers in which the different compositions of varying dimers will determine the resulting differential transcriptional and biological functions [176]. The activation of AP-1 by different stimuli, such as cytokines, growth factors, and oncogenic stimuli leads to uncontrolled cellular proliferation and prevent the cancer cells from undergoing apoptosis [177,178]. Primarily, AP-1 pathway is activated through the combination of signalling events mostly by mitogen-activated protein kinases (MAPKs) which consist of; the extracellular-signal regulated kinases (ERKs), the c-JUN amino-terminal kinase (JNKs) and p38 family of kinases which directly activates the transcription of Jun and Fos [179]. Besides, the activation of AP-1 is often associated with high levels of NF-κB which also implicated in tumorigenesis [180]. 

Constitutive AP-1 activity in PCa disease is associated with poor clinical outcomes through modulating cancer-related genes expression involved in inflammation, cell proliferation, neoplastic transformation and metastasis [181,182,183]. Studies have reported that the correlation between Fos (Fra-1) and Jun family (c-Jun) proteins has been associated with tumour growth in multiple types of cancer. In PCa, on the other hand, Jun protein family was reported have played a major role in controlling cell growth and survival [184]. Other studies demonstrated that Jun family (JunD), along with Fos family proteins (Fra-1 and Fra-2) are also implicated in PCa proliferation and conferring the protection against radiation-induced cell death [185]. Over-expression of c-Jun in ADPC (LNCaP) cells has shown to increase cell proliferation and reduce of cell death [186]. Other studies reported that the increased cytoplasmic phosphorylated ATF proteins family (ATF2) in PCa compared to normal prostate cells suggest that altered localisation of ATF2 may contribute to clinical progression of PCa [187]. Meanwhile, the activation of the member of Fos family (Fra-1) and Jun family (c-Jun) proteins have associated with the progression to AIPC state [182,188]. Elevated levels of Jun and Fos proteins in mouse models of PCa was also correlated with prostate tumorigenesis, whereas the levels of Jun proteins alone is correlated with disease recurrence [189]. It was also demonstrated that upregulation of Raf-1 promotes the correlation with HER2/Raf-1/AP-1 axis, particularly via modulation of AP-1, resulting in the development of AIPC and early relapse [190]. Other studies reported that AP-1 is a mediator of epidermal growth factor receptor (EGF-R), PI3K and MAPK/ERK pathways whereby the inhibitors of these pathways are able to suppress expression of several AP-1 subunits during disease progression and also sensitises the radiation response of AIPC (PC-3 and DU145) cells [183]. 

Curcumin was shown to inhibit the expression of AP-1 in multiple types of cancer such as astroglioma, colon, cervical and PCa [169]. As shown in Figure 4c, curcumin inhibits the activation of AP-1 via a direct interaction with AP-1 DNA-binding motif [191,192]. Curcumin also inhibits the activation of AP-1 as well as JNK which was induced by tumour promoters and carcinogens [193,194]. Curcumin suppresses tumour progression of AP-1 in both ADPC (PC-3) and AIPC (LNCaP) cells, which indicated by the reduced colony forming ability in soft agar [92,103]. Other studies reported that curcumin exhibits its anti-cancer effects by significantly impeding AP-1 protein in AIPC (PC-3) cells [108]. Besides, curcumin treatment is able to promote cell cycle arrest and apoptosis in ADPC (LNCaP) cells by regulating the level of c-Jun proteins, an important member of the AP-1 complex which is primarily activated via phosphorylation by the c-Jun amino terminal kinase (JNK) [116,117]. Furthermore, curcumin has shown the ability to reduce cell proliferation and migration of ADPC (LNCaP) cells by suppressing the activation of AP-1 that are stimulated by hydrogen peroxide [118]. Curcumin treatment is also able to modulate AP-1 activity in AIPC (DU145) cells which leads to the disruption of the survival pathways by sensitising the cells, thus potentiating TNF-induced apoptosis [92]. These findings indicate that curcumin may appear to be a potent AP-1 inhibitor agent that may act as a therapeutic agent for PCa therapy.

### 4.4. Phosphatidylinositol 3-kinases/the Serine/threonine kinase (PI3K/Akt)

PI3K/Akt/mTOR signal transduction pathway is involved in the regulation of multiple cellular physiological processes by activating downstream corresponding effector molecules, which serves an important role in cell survival and growth. Dysregulation of downstream kinases in PI3K/Akt/mTOR pathway are common in many types of cancer [195]. PI3K, a heterodimeric enzyme is typically initiated by the binding of a growth factor such as EGFR, and eventually results in the downstream activation of PI3K signal transduction [196]. Upon activation, PI3K phosphorylates membrane-bound phosphatidylinositol-(4,5)-bisphosphate (PIP_2_) to phosphatidylinositol-(3,4,5)-trisphosphate (PIP_3_), which subsequently acts as a secondary messenger triggering the downstream signalling events. Following this, PIP_3_ recruits a subset of signalling proteins to pleckstrin homology (PH) domain of Akt, which in turn activates Akt, which an important cell growth regulator. Activated Akt then phosphorylates various downstream targets involved many biological functions including cell survival, angiogenesis, metastasis and therapy resistance [197,198]. PTEN, a well characterised negative regulator of PI3K action antagonises the Akt activation by dephosphorylating PIP_3_ to PIP_2_, thereby opposing PI3K activity and subsequently inhibiting cell proliferation [199].

The PI3K signalling pathway plays an important role in PCa progression and the development of castration resistance. In fact, it is one of the most commonly altered signalling pathway occurred in PCa [200]. Excessive activation of PI3K/Akt/mTOR pathway has been identified in early and advanced stage of PCa as a result of the loss of function of PTEN, normally through mutations [201,202,203,204]. Constitutively activated PI3K/Akt/mTOR pathway in PCa is accompanied with the loss of PTEN functions and an increased of AKT-1 phosphorylation [205,206]. The loss of PTEN expression in PCa promotes the acceleration of the disease progression and also correlated with higher Gleason score, advanced stage, and poor prognosis among patients [204]. Furthermore, activation of the PI3K pathway is also associated with ADT resistance and is commonly occur during the progression from ADPC to AIPC [207,208]. Constitutive activation of PI3K pathway has also been observed in 20-40% of primary PCa and 60% of AIPC [209]. Therefore, the aberrance of the downstream targets of this pathway are linked with cell survival and proliferation, invasion, metastasis as well as therapy resistance in PCa [210,211].

Curcumin generally targets various signalling pathways including PI3K/Akt pathway which leads to inhibition of tumour growth and disease progression in PCa (Figure 4d) [56,212]. It was shown that curcumin exhibits anti-cancer effect in several tumour models through regulating PI3K/Akt/mTOR pathway whereby it suppresses the Akt activation along with downstream targets, mTOR [213]. In response to curcumin treated ADPC (LNCaP) cells, PI3K/Akt/mTOR pathway was downregulated which leads to apoptosis and induction of cell cycle arrest [119]. Other studies have reported that curcumin induced apoptosis not only in ADPC (LNCaP) and but also in AIPC (DU145 and PC-3) cells through the downregulation of PI3K p110 and p85 subunits, and phosphorylation of Ser 473 Akt. This has increased the permeabilisation of the mitochondrial outer membrane and trigger the release of mitochondrial proteins into the cytosol [120]. Besides, curcumin also inhibited PI3K activity in AIPC (PC-3) cells, mediated by changes in the phosphorylation status of Akt [96]. Curcumin also exhibited chemo- and radio-sensitising effects by downregulating the murine double minute 2 (MDM2) oncogene through the PI3K/mTOR/ETS2 pathway [96]. Additional evidence detailing the mode of action of curcumin in inhibiting the phosphorylation of Akt, mTOR, and their downstream substrates in AIPC (PC-3) cells, were directly affect the downstream of PI3K and PDK1 activities [121]. Furthermore, few studies were also demonstrated that curcumin also suppresses the cell proliferation in AIPC (DU145) cells by inhibiting Akt/mTOR signalling [121,122]. These finding suggest that curcumin is able to reduce the cancer cells viability and induced apoptosis by significantly inhibiting the PI3K/Akt/mTOR pathway which eventually may improve the PCa therapy.

### 4.5. Bcl-2 family

The apoptosis process is mainly regulated by B-cell lymphoma 2 (Bcl-2) family proteins, which consist of anti-apoptotic (e.g., XIAP, Bcl-2, Bcl-xL) and pro-apoptotic (e.g., Bim, Bax, Bak, Bid, Puma and Noxa) proteins. The fate of a cell depends on the ratio of apoptotic proteins either by stimulation of the pro-apoptotic molecules or by inhibition of the anti-apoptotic molecules. Among the anti-apoptotic proteins, Bcl-2 protein plays a pivotal role in cell survival activities as well as chemo-resistance which frequently dysregulated in many types of cancers, including PCa [96,214]. Bcl-2 also responsible in the progression of ADPC to AIPC [215,216]. Meanwhile, studies also reported that the over-expression of Bcl-xL was associated with higher Gleason grade and the onset of AIPC [217]. An increased level of Bcl-2 expression protects PCa cells from undergoing apoptosis through association with PTEN loss, p53 inactivation, PI3K/Akt phosphorylation, and the activation of RTK/STAT3/NF-κB, Ras/Raf1/MEK/ERK pathways and autophagy proteins (e.g., Beclin1 and AMBRA1) [218]. 

Curcumin has been shown to induce apoptotic activity in prostate cancerous cells by regulating various cell-signalling pathways via extrinsic or intrinsic pathway as illustrated in Figure 4e [219,220]. It was reported that curcumin downregulates Bcl-2, Bcl-xL, and XIAP and upregulates the expression of p53, Bax, Bak, PUMA, Noxa, and Bim proteins which is attributed to the activation of caspases, cleavage of PARP and eventually cell death [85,92,93,120]. Curcumin also mediates apoptosis by affecting apoptotic-related molecules such as EGFR, erbB2, Hedgehog, AR, PI3K/Akt, NF-κB, Bcl-2, Bcl-xl, AP-1, and TMPRSS2-ERG fusion protein [120,126,221]. 

Pre-clinical and clinical studies have reported that curcumin capable to induce apoptosis in ADPC, AIPC, and metastatic PCa either via intrinsic or extrinsic pathways [44,93]. Previous study has reported that curcumin induce apoptosis in ADPC (LNCaP) cells in concentration-dependent manner [120]. Curcumin initiates the ADPC (LNCaP) cells to undergo apoptosis by translocation of Bax and p53 to mitochondria, the production of ROS, the reduction in mitochondrial membrane potential, the release of mitochondrial proteins (cytochrome c, Smac/DIABLO and Omi/HtrA2), and activation of caspase-3 [120,123]. Furthermore, nude mice implanted heterotopically with (ADPC) LNCaP cells has depicted the induction of apoptosis potentially attributable to curcumin [94]. Moreover, curcumin inhibits cell growth and induce apoptosis in both ADPC and AIPC cells but has no effect on normal human prostate epithelial cells [120]. 

Meanwhile, curcumin treatment also triggers apoptosis in AIPC by inducing caspase-3 activity in a dose-dependent manner [120]. A study demonstrated that upon curcumin treatment in AIPC (PC-3 and DU145) cells, the cells undergo apoptosis and autophagy which mediated by cell cycle arrest at G2/M phase [124]. Curcumin-treated AIPC (DU145) cells has revealed significant suppression of Bcl-2 expression, while procaspase-3 is activated simultaneously [125]. The treatment of curcumin in AIPC (PC-3) nude mice model displays apoptosis process by upregulating Bax and downregulating Bcl-2, and also regulating the mitochondrial outer membrane permeability [126]. Other studies demonstrated that curcumin triggers apoptosis in AIPC (PC-3) cells which was associated with mitochondria damage and cell ceramide accumulation resulting in PC-3 cells apoptosis [127]. Curcumin treated AIPC (PC-3) cells has triggered an increased apoptotic cell death which mediated by caspase activation and the loss of mitochondrial membrane integrity [128]. Furthermore, since expression of anti-apoptotic Bcl-2, Bcl-xL, and XIAP is regulated by NF-κB, the inhibition of NF-κB and NF-κB-regulated anti-apoptotic genes products through suppression of Akt by curcumin in AIPC (PC-3) cells significantly induce the apoptosis proteins [113]. On the other hand, curcumin downregulates MDM2 oncogene, which are negative regulators of the p53 thus allowing PCa cells to undergo apoptosis [96]. Curcumin also mediates apoptosis through cell cycle arrest due to induced expression of p16, p21, and p27; increased the ER stress; and by downregulating MDM2 [97,104]. Therefore, curcumin is able to trigger apoptosis by targeting Bcl-2 family which may represent an important strategy in the development of PCa treatment.

### 4.6. Cyclin D1

Accelerated proliferation of malignant cells may result the aberrant activities of cell cycle proteins and the imbalance of cell cycle checkpoints [222]. Cyclin-dependent kinases (CDKs), are the key intracellular mediators that regulate the initiation, progression and completion of cell cycle division [223]. CDKs act as the engine that drives cell cycle progression activated by binding to cyclins [224]. CDK/cyclin complex is tightly regulated by cyclin-dependent kinase inhibitors (CDIs), a negative regulator of CDKs which halt the cell cycle progression under unfavourable conditions [225]. Dysregulation expression of cyclins and CDIs such as p21 and p27 effects the cyclin/CDK complexes activity which eventually leads to abnormal cell proliferation and tumour growth [226]. 

Cyclin D1, the most predominantly cyclins associated with carcinogenesis, forms active complexes by binding to CDK4 and/or CDK6, then phosphorylates the retinoblastoma protein (Rb), which consequently governing the progression from G1 to S phase [227,228]. Over-expression of cyclin D1 shortens the G1-S transition, thus promoting tumorigenesis and cancer recurrence in diverse human cancers [229,230]. In PCa, cyclin D1 expression is upregulated and correlated with high-grade Gleason score [231]. Cyclin D1 also leads to transformation of androgen-independent state through the upregulation of MDM2 [232]. Besides, highly expressed cyclin D1 in AIPC cells acquired radio-resistance properties and accelerates the relapse of the disease [233,234]. 

Curcumin has shown the ability to modulate cell cycle regulatory molecules, conferring anti-proliferation and induction of apoptosis in cancer cells as illustrated in Figure 4f [228,235]. Inhibitory effect of curcumin in LNCaP (ADPC) cells was shown through cell cycle arrest indicated by downregulation of cyclin D1 expression via inhibition of CDK4-mediated phosphorylation of Rb protein [92]. Curcumin has also shown the ability to induce cell cycle arrest at G1/S, followed by apoptosis when treated in (ADPC) LNCaP and AIPC (PC-3) treated cells [97]. Meanwhile, other studies have reported that curcumin promotes cell cycle arrest at G2/M phase in both type of PCa cell lines [129]. The cell cycle arrest activity was attributed to the inhibition of cyclin E and cyclin D1 expression, and hyperphosphorylation of Rb protein. Apart from that, curcumin induces the expression of several CDIs proteins such as p16, p21 and p27 which also leads to inhibition of the cell cycle progression [97]. Other findings demonstrated that curcumin induces G0/G1 arrest in AIPC (DU145) treated cells by suppression of cyclin D1 and CDK2 expression, while upregulating p21 and p27 [125]. In addition, curcumin suppresses cell proliferation in ADPC (LNCaP) xenograft model by downregulating cyclin D1 and upregulating TRAIL-R1/DR4, TRAIL-R2/DR5, Bax, Bak, p21 and p27 proteins [130]. Curcumin also downregulates cyclin D1 expression through inhibition of ß-catenin accumulation in ADPC (LNCaP) cells and xenograft model [102,106].

Cyclin D1 activity is also mediated by extracellular signals and a variety of growth factors, where EGF acts as the main mediator [236,237,238]. In PCa, EGF regulates cell proliferation partially through regulation of cyclin D1, whereby EGFR translocates to the nucleus and act as a vector for cyclin D1 [239,240]. Over-expression of EGFR family, especially c-erbB-1 and c-erbB-2 are frequently occurred in multiple types of cancers including PCa [241,242]. Curcumin acts as a potent inhibitor for EGFR and ERBB2 receptor when treated in ADPC (LNCaP) cells, inhibiting ligand-induced activation for EGFR and its intrinsic tyrosine kinase activity associated with the downregulation of cyclin D1 [131]. Curcumin is also reported to have an inhibitory effect on EGFR phosphorylation in AIPC (PC-3) cells [132]. Hence, it is well-established that curcumin inhibits EGFR signalling pathway as well as cyclin D1 expression which are implicated in PCa [56]. The approach of targeting EGF and EGFR in addition to the regulation of CDK-cyclin families especially cyclin D1 by curcumin could be a promising strategy for the treatment of PCa.

### 4.7. Wnt/ß-catenin

Wingless (Wnt)/ß-catenin signalling pathway is one of the vital mechanisms responsible for the cell proliferation and tissue homeostasis maintenance [243]. When in inactive state, cytoplasmic β-catenin is sequestered in a multiprotein “degradation complex” which composed of scaffolding Axin proteins, glycogen synthase kinase 3ß (GSK-3β), casein kinase 1α (CK1α), adenomatous polyposis coli gene product (APC) and protein phosphatase 2A (PP2A) [244,245]. After sequential phosphorylation by CK1α and GSK-3β, the phosphorylated β-catenin undergoes ubiquitination and degradation by proteasomes thus maintaining the inactivity of this pathway [246]. On the contrary, accumulation of the extracellular Wnt ligands, association of Axin with phosphorylated LRP5/6 (lipoprotein receptor-related protein 5/6) and recruitment of phosphorylated DVL (dishevelled) to FZD (frizzled) lead to the dissociation of the “destruction complex”. This dissociation allows translocation of β-catenin into the nucleus which forms an active complex with T-cell factor/lymphoid enhancing factor (TCF/LEF), and consequently activates the target genes which involved in cell growth including c-myc, CCND1, survivin, E-cadherin, COX-2, MMP, and VEGF [247,248].

The dysregulation of Wnt/β-catenin pathway and its downstream is a common event in multiple malignancies, including PCa [249,250,251,252,253,254]. The aberrance of this pathway leads to a highly aggressive disease with poor prognosis in PCa [255]. Also, a study has demonstrated that over-expression of Wnt/β-catenin pathway in AIPC (PC-3 and DU145) cells indicates the importance of this pathway in the development and progression of PCa [256]. A recent study on sequencing of PCa genomes reveals that mutations in major components of the Wnt/β-catenin pathway are frequently occurred in AIPC cells [257]. 

On the other hand, curcumin is able to modulate the conical Wnt/β-catenin pathway in PCa as illustrated in Figure 4g [258,259]. Curcumin has shown an impact on cell growth inhibition in ADPC (LNCaP) cells by reducing the level TCF-4, CBP, and p300 proteins that leads to the decrease of ß-catenin/TCF-4 transcriptional activity, which subsequently decreases the expression of β-catenin target genes [70,133]. Curcumin has also shown the ability to suppress the Wnt/ß-catenin signalling pathway treated in ADPC (LNCaP) cells [102,106].

In addition, an interplay between AR and Wnt/β-catenin pathway enhanced the androgen-mediated transcription which leads to prostate tumorigenesis [260,261]. The interaction of β-catenin with AR promotes transcriptional activity in ADPC (LNCaP) cells, which suggesting a possible mechanism of crosstalk between Wnt and androgen signalling pathways [262]. Curcumin is able to control cell proliferation and angiogenesis by inducing the degradation of β-catenin through the regulation of downstream molecules of Wnt/β-catenin pathway. As β-catenin is coupled with AR as a potent coactivator, curcumin treatment downregulates the AR expression as well as reducing the intracellular accumulation and nuclear translocation of β-catenin [102]. 

A phosphorylated GSK-3β can stabilise LRP5/6 that promotes β-catenin signalling, therefore the inhibition of GSK-3β may suppress β-catenin–mediated gene expression [263]. Curcumin affects the cell proliferation in ADPC (LNCaP) cells by suppressing the GSK-3β phosphorylation thus inducing the degradation of β-catenin. Consequently, curcumin may serve not only to prevent accumulation of β-catenin, but also to degrade target substance such as cyclin D1 and c-myc [102]. The suppression of ß-catenin transcriptional activity by curcumin is also mediated through the activation of PDK1 [264]. In comparison to normal cells, PDK1 activity is much less expressed in PCa cells, which triggers the initiation of prostate carcinogenesis [265,266]. On the other hand, curcumin activates PDK1 activity, resulting in attenuation of nuclear β-catenin/TCF transcription activity. Such mechanism modulates the phosphorylation, and translocates the nuclear ß-catenin out from the nucleus and enriches membrane localisation of β-catenin [267]. Taken together, we can conclude that curcumin has the ability to modulate Wnt/ß-catenin pathway and regulate the activation of AR, GSK-3β and PDK1 and it is therefore suggested that curcumin may act as a potential therapeutic agent in targeting PCa.

### 4.8. Role of MicroRNA (MiRNA)

MicroRNAs (miRNAs) are small non-coding RNAs that regulate gene expression post-transcriptionally and aberrantly expressed in many types of cancers, including PCa [268,269]. Dysregulation of miRNA in cancer are normally caused by genetic alterations; amplifications, deletion or mutations, or abnormal transcriptional control of miRNAs; or defects in components of the miRNA biogenesis machinery [270,271]. In PCa, aberrant expression of miRNAs contributes to cellular growth alteration, metastasis and development of AIPC by regulating the expressions and functions of their target genes. Numerous studies in in vitro and in vivo have reported that aberrant expression of miRNAs are associated with the disease progression in both ADPC and AIPC [272,273]. Curcumin has been reported is able to regulate various miRNA expression profile in many types of cancers by upregulating tumour suppressive miRNAs while downregulating oncogenic miRNAs in order to exert its anti-cancer properties [274,275]. Curcumin inhibits cancer cell proliferation and promotes apoptosis through upregulating a set of tumour suppressor miRNAs, such as miR-15a, miR-34a, miR-181b, miR-186, miR-192-5p, and miR-215, or downregulating numerous onco-miRNAs, like miR-19, miR-21, and miR-208 [276].

Curcumin has shown the ability to inhibit cell proliferation and migration of AIPC (DU145) cells by upregulating the expression of miR-143, which could be attenuated by transfection with anti-miR-143. In PCa, mir-143 expression is predominantly expressed, indicating an association with the PCa development [277]. Similar to curcumin, overexpression of miR-143 downregulates the expression of phosphoglycerate kinase-1 (PGK1), which is associated with the aggressiveness of PCa. Curcumin is also able to increase the level of forkhead box D3 (FOXD3), a transcriptional factor for miR-143 [34,134]. The ectopic expression of FOXD3 synergized with curcumin in upregulating the expression of miR-143 resulting in suppression of tumour progression [278]. It was also shown that curcumin is able to restore miR-143/miR-145 cluster expression in ADPC (LNCaP) and AIPC (PC-3 and DU145) cells via hypomethylation. MiR-143/miR-145 cluster is widely recognized as a tumour suppressor miRNA and frequently aberrated in PCa. Downregulation of miR-143/miR-145 cluster is associated with an increased cell proliferation and migration [279,280]. The upregulation of miR-143/miR-145 cluster expression by curcumin adversely inhibits the cell migration, cell proliferation and invasion by targeting Golgi membrane protein 1 (GOLM1) and hexokinase-2 (HK2) [135,136]. Moreover, restoration of miR-143/miR-145 cluster may suppress stem cell characteristics of PCa cells via downregulating CD133, CD44, Oct4, c-Myc and Klf4. Both miR-143 and curcumin is able to sensitize AIPC (PC-3 and DU145) cells to radiation via downregulation of autophagy-related protein 2 homolog B (ATG2B), which enhanced the radiation-induced apoptosis in PCa cells [281]. Following this, an approach investigating the interaction between miRNAs and their target genes could be a potential therapeutic strategy in the treatment of PCa.

## 5. Clinical Trials

A number of clinical trials have shown the efficacy of curcumin as anti-cancer agent in several types of cancer including pancreatic, colorectal, cervical, oral and breast cancer [282,283]. However, clinical studies documenting the inhibitory effects of curcumin in PCa is scarce. Almost all of the existing clinical studies report the effects of curcumin towards PCa only as an adjuvant therapy, either in radiotherapy, hormonal or chemotherapeutic interventions but none of them are reporting curcumin alone as the main therapeutic agent. 

One of the completed studies reports the anti-cancer effects of curcumin in PCa patients that undergo intermittent androgen deprivation (IAD) (clinicaltrials.gov code NCT03211104) [284]. During the off-treatment of IAD, results have shown that oral intake of curcumin for six months duration is able to suppress the PSA levels in patients [285]. In another study, combination of curcumin with the standard chemotherapy agent, docetaxel and prednisone in patients with castration-resistance PCa demonstrated that curcumin enhances the efficacy of the treatment by increasing the response rate, tolerability and patient acceptability [286]. There is another clinical study which analyses the effects of curcumin as a radio-sensitising and radio-protective agent in PCa patients (clinicaltrials.gov code NCT01917890) [287]. The results showed that curcumin improves antioxidant status in PCa patients who received radiotherapy [288]. Curcumin supplement can improve lower urinary tract symptoms in PCa patients who undergo radiotherapy [289]. Another clinical study was designed to assess the curcumin supplement, together with isoflavones on the serum PSA levels, and given to patients who had prostate biopsy due to elevated PSA levels but were not found to have PCa. After six months of oral intake of isoflavones and curcumin, a significantly decreased of serum PSA levels was observed [290]. 

At the moment, there are two ongoing clinical studies which focused on the effects of curcumin towards PCa. One of the studies is investigating the potential of adjuvant use of curcumin after prostatectomy in improving recurrence-free survival administered in PCa patients (clinicaltrials.gov code NCT02064673) [291]. The other study, which currently in recruiting phase is evaluating the potential of curcumin in reducing the risk of PCa progression in low-risk men which undergoing active surveillance (clinicaltrials.gov code NCT03769766) [292].Based on these positive outcomes of curcumin as an adjuvant therapy, therefore it is suggested that clinical studies of curcumin alone are warranted in order to implement curcumin as a standard treatment for PCa. The suggested studies may extend the current understanding of curcumin’s efficacy and mechanism of actions against PCa. Table 2 depicts the summarized information for completed and ongoing clinical trials on the effects of curcumin in PCa.

## 6. Conclusions and Future Perspectives

Despite the advancement in PCa treatment modalities, there is still no decline in incidence and mortality rates of PCa. The available treatments for PCa are more to palliative, where a prolonged intake may cause unfavourable effects. Curcumin is shown to have the ability to delay the early onset of PCa and inhibits progression of the disease from ADPC to AIPC state by modulating multiple key signalling pathways; AR, AP-1, PI3K/Akt/mTOR, Wnt/ß-catenin, and several molecular targets including NF-κB, Bcl-2 and cyclin D1. In spite of its widely reported health benefits, the use of curcumin is hampered by its poor bioavailability which limits its clinical application. Several strategies have been developed to address these limitations, including designing new structural analogues and improving the delivery system by encapsulation of curcumin in the forms of nanoparticles, liposomal encapsulation, and emulsions, therefore maximising the potential of curcumin in combating PCa [66]. Further pre-clinical and clinical studies are required to better understand in terms of mechanism of action of curcumin, enhanced bioavailability, safety, dose efficacy and stability in order to translate curcumin as a drug candidate to treat PCa. 

## Figures and Tables

**Table 1:** Molecular mechanism targeted by curcumin in vivo and in vitro against androgen-dependent and androgen-independent prostate cancer.

Molecular Target	Cell Lines/In-Vivo	Molecular Mechanism Modulated by Curcumin	Reference
**Androgen receptor (AR)**	LNCaP	Downregulated AR expression via limiting the binding activity to the ARE of the PSA gene	[100,101]
	LNCaP	Inhibited cell proliferation and growth via modulation of AR and its signalling pathway	[102]
	LNCaP	Inhibited tumour growth and suppressed the PSA level by the activation of AR and interleukin-6	[100]
	LNCaP & PC-3	Downregulated AR expression and transcriptional activity	[103]
	LNCaP & TRAMP model	Decreased intracellular prostate testosterone level	[102]
	PC-3	Reduced AR availability by altering the over-expressed heat shock protein (Hsp90)	[104]
	LNCaP xenograft	Delayed the tumour growth and suppressed AR expression	[105]
	LNCaP xenografts	Inhibited AR through the modulation of Wnt/ß-catenin signalling	[106]
	LNCaP & PC-3	Downregulated the activation of AR-related cofactors	[100,103]
	LNCaP	Initiated apoptosis and downregulated the AR activity	[93]
	LNCaP	Reduced NKX3.1 and AR expression	[107]
**NF-κB**	LNCaP & DU145	Suppress NF-κB expression thus abrogates their survival mechanisms	[92]
	PC-3	Inhibited cell proliferation and induced apoptosis via suppressed NF-κB expression	[108]
	LNCaP	Suppressed cell proliferation through downregulation of cyclin D1 by inhibiting NF-κB	[92]
	PC-3	Enhanced cytotoxicity by suppressed constitutional and TNF-α-induced NF-κB activation	[109]
	PC-3 mouse model	Prevented metastasis by downregulating CXCL-1 and -2 by targeting NF-κB signalling	[110]
	LNCaP, PC-3 & DU145	Sensitised PCa cells towards TRAIL-induced apoptosis	[111,112]
	LNCaP	Initiated apoptosis by effecting intrinsic and extrinsic pathways	[111]
	LNCaP	Induced cytotoxicity by inhibiting phosphorylation and degradation of IκBα	[111]
	LNCaP & PC-3	Combination of TRAIL inhibits Akt-regulated NF-κB and NF-κB-dependent anti-apoptotic proteins	[113]
	LNCaP & PC-3	Chemosensitization to TRAIL therapy inhibited a constitutively active NF-κB, AP-1 and active anti-apoptotic Akt (p-Akt)	[112,113,114]
	PC-3 xenograft model	Combination with TRAIL inhibition the growth indicated by NF-κB and AP-1 inhibition	[115]
**Activating protein-1 (AP-1)**	PC-3 & LNCaP	Suppressed tumour progression of AP-1, which indicated by the reduced colony forming ability in soft agar	[92,103]
	PC-3	Exhibited anti-cancer effects by impeding AP-1 protein	[108]
	LNCaP	Promoted cell cycle arrest and apoptosis by regulating the level of c-Jun proteins, which is activated via phosphorylation by the c-Jun amino terminal kinase (JNK)	[116,117]
	LNCaP	Reduced cell proliferation and migration by suppressing the activation of AP-1 which stimulated by hydrogen peroxide	[118]
	DU145	Disruption of the survival pathways by sensitising the cells, thus potentiating TNF-induced apoptosis	[92]
**PI3K/Akt**	LNCaP	Apoptosis and cell cycle arrest by downregulating PI3K/Akt/mTOR pathway	[119]
	LNCaP, DU145 & PC-3	Apoptosis by downregulating PI3K p110 and p85 subunits, and phosphorylation of Ser 473 Akt.	[120]
	PC-3	Decreased PI3K activity mediated by changes in the phosphorylation status of Akt	[96]
	PC-3	Inhibited the phosphorylation of Akt, mTOR, and their downstream substrates which directly affect the downstream of PI3K and PDK1 activities	[121]
	DU145	Suppressed cell proliferation by inhibiting Akt/mTOR signalling	[121,122]
**Bcl-2 family**	LNCaP	Induced apoptosis in concentration-dependent manner	[120]
	LNCaP	Initiated apoptosis by translocation of Bax and p53 to mitochondria, the production of ROS, the release of mitochondrial proteins, and activation of caspase-3	[120,123]
	LNCaP implanted nude mice	Induced apoptosis	[94]
	PC-3 & DU145	Apoptosis and autophagy, mediated by cell cycle arrest at G2/M phase	[124]
	DU145	Induced apoptosis by suppressing the Bcl-2 expression, while activating procaspase-3 simultaneously	[125]
	PC-3 nude mice model	Apoptosis by upregulating Bax and downregulating Bcl-2, and regulating the mitochondrial outer membrane permeability	[126]
	PC-3	Apoptosis by mitochondria damage and cell ceramide accumulation	[127]
	PC-3	Increased apoptotic cell death mediated by caspase activation and the loss of mitochondrial membrane integrity	[128]
	PC-3	Induced the apoptosis proteins by inhibition of NF-κB and NF-κB-regulated anti-apoptotic genes products through suppression of Akt	[113]
**Cyclin D1**	LNCaP	Inhibited growth through cell cycle arrest indicated by downregulation of cyclin D1 expression via inhibition of CDK4-mediated phosphorylation of Rb protein	[92]
	LNCaP & PC-3	Induced cell cycle arrest at G1/S, followed by apoptosis	[97]
	LNCaP & PC-3	Induced cell cycle arrest at G2/M phase	[129]
	DU145	Induced G0/G1 arrest by suppression of cyclin D1 and CDK2 expression, while upregulating p21 and p27	[125]
	LNCaP xenograft model	Suppressed cell proliferation by downregulating cyclin D1 and upregulating TRAIL-R1/DR4, TRAIL-R2/DR5, Bax, Bak, p21 and p27 proteins	[130].
	LNCaP & LNCaP xenograft model	Downregulated cyclin D1 expression through inhibition of ß-catenin accumulation	[102,106].
	LNCaP	Inhibiting ligand-induced activation for EGFR and its intrinsic tyrosine kinase activity associated with cyclin D1 downregulation	[131]
	PC-3	Inhibited the EGFR phosphorylation	[132]
**Wnt/ß -catenin**	LNCaP	Inhibited cell growth by reducing the level TCF-4, CBP, and p300 proteins that leads to the decrease of ß-catenin/TCF-4 transcriptional activity thus decreased β-catenin expression	[70,133]
	LNCaP	Inhibited cancer growth by suppressing the Wnt/ß-catenin signalling pathway	[102,106]
	LNCaP	Inhibited cell proliferation by suppressing the GSK-3β phosphorylation thus inducing the degradation of β-catenin	[102]
**MiRNA**	DU145	Inhibited cancer growth and migration by upregulating the expression of miR-143	[34,134]
	LNCaP, PC-3 & DU145	Inhibited cell proliferation and migration by restoring miR-143/miR-145 cluster expression	[135,136]

Abbreviations: Androgen receptor (AR) signalling, Activating protein-1 (AP-1), Phosphatidylinositol 3-kinases/the serine/threonine kinase (PI3K/Akt/mTOR), Wingless (Wnt)/ß-catenin signalling, and molecular targets; Nuclear factor kappa-B (NF-κB), B-cell lymphoma 2 (Bcl-2) and Cyclin D1.

**Table 2:** Completed and ongoing clinical trials on the effects of curcumin in prostate cancer.

Intervention	Study	Status	Identifier Number/ Reference
Curcumin	Effects on PCa patients that undergo intermittent androgen deprivation (IAD)	Completed	NCT03211104/ [66]
Curcumin, Docetaxel & Prednisone	Combination with standard chemotherapy agents, docetaxel and prednisone in patients with castration-resistance PCa	Completed	*[286]
Curcumin	Radiosensitizing and radioprotective effects in PCa patients	Completed	NCT01917890/ [287]
Curcumin & Isoflavones	Combination with isoflavones who had prostate biopsy due to elevated PSA levels but do not have PCa	Completed	* [290]
Curcumin	Adjuvant use of curcumin after prostatectomy in improving recurrence-free survival for PCa patients	Recruiting	NCT02064673/ [291]
Curcumin	Effects on prevention progression of low-risk PCa under active surveillance	Recruiting	NCT03769766/ [292]

* NCT number National Clinical Trial (NCT) Identifier not shown.
